# Diphtheria

**Published:** 1860-01

**Authors:** W. Godfrey Dyas

**Affiliations:** Chicago


### ARTICLE 2.

### DIPHTHERIA.

BY W. GODFREY DYAS, M. D., CHICAGO.

[Continued from Page 673 Vol. 2.]

Perhaps no part of France suffered to a greater extent from Diphtheria, than Boulogne. Here a large proportion of the inhabitants is British, and from among these the pestilence unsparingly selected its victims. Within two years—from 1855 to 1857—366 persons succumbed to its virulence; and there, as elsewhere, the same insidious approach—the same pseudo-membranous patch in the fauces—and the same prostration, were its attendants. As in Paris, so in Boulogne, the extension of the false membrane was rather towards the nares than the larynx; but the onset, in many instances, was more frequently marked by nausea—a symptom that soon came to have a fearful significance. This nausea was succeeded by vomiting, on which supervened other well-known signs, which too truly indicated that the system was already unequally committed to a struggle with an enemy that seldom failed to come off victorious, and to oppose which, the resources of art had too often proved unavailing. Occasionally, the disease set in with convulsions, which indicated peculiar danger. When the membranous exudation rapidly extended to the posterior nares, the prognosis in every such case was unfavorable. Meningitis and Gastro-Enteritis were no uncommon complications. Often the enlargement of the cervical and submaxillary glands preceded the deposition of false membrane. It is remarkable that in no two places was there a perfect resemblance either in the grouping of the symptoms—in their order of succession—or in the degree of their individual development. Still, in all, the essential features of the epidemic were alike. In Avignon, in 1853, out of a force of 1686 soldiers, it attacked 195. It exhibited a decided predilection for children, 18 per cent. of whom were seized; whilst of adults, but four per cent. were sufferers. Here the almost universally fatal symptom was a sense of constriction in the larynx. M. Lespiau (Relation d'une Epidemic Diptheritique qui a sevi sur le 75e de ligne) observed that but one of those who had this symptom recovered. In Nievere the disease did not attack the larynx. In the Haute Marne, the nasal fossa were the principal seat of its local manifestation; and we are told by Jobert that the epistaxis consequent on this, materially contributed to the fatal issue. On the other hand, in the neighborhood of the Pyrenees, the disease, as in Tours in 1818, chiefly destroyed by its extension to the larynx. How slowly the disease progressed from the time it appeared in that year among the soldiers of the legion of La Vendee until its arrival in 1855 in Boulogne! How capricious in its habits, and how fluctuating in its character! On its reaching a district previously unassailed, no opinion of its probable course, or of its amount of malignancy, could be formed from any experience of these acquired elsewhere. Having arrived at Boulogne, which maintains uninterrupted intercourse with England, the breadth of the English Channel proved, as might be expected, but a feeble barrier to the transmission of the disease to that country. Accordingly, early in the year 1857, we have the first distinct intimation of its arrival in England. It then appeared in Kent, from which it extended to Essex. Its progress was more especially in the direction of the eastern countries. Yet it was not limited to these. Almost simultaneously, we hear of its being in Cornwall. During the Spring and Summer, it seemed not to have the virulence it displayed in France; but the Winter of the same year witnessed a progress as devastating as any experienced in the latter country. Not confining its attacks to such as had been enfeebled, it indiscriminately seized on the cachectic and robust. In some districts, it was comparatively mild. In Dudley in Staffordshire, the deaths were not in proportion to the number attacked. It was frequently complicated. In Gainsborough, Canterbury, Ashton under Lyne, Eastry, Freeham, Twickenham, it was associated with scarlet fever; with whooping-cough in Macclesfield, Congleton and Nantwich; with gastro-enteritis in Lincolnshire; with gastric fever at Beccles; with small pox in Lancashire; with measles at Acton, where out of twenty-five deaths eleven were due to diphtheria complicated with measles. Small pox proved a fearful complication, and largely contributed to the mortality. But in no part of England was diphtheria more virulent in form than in Norfolk. "In the district of Tunstead, six children of one man at Swafield, were carried off within five weeks, being all the family, with the exception of one infant at the breast." (*Report of the Lancet Sanitary Commission.*)

The epidemic in Essex, if not more severe in form, was perhaps more general and extensive in its attack than elsewhere. In the words of the "Report," "the affection (in Essex) was almost universal." But taking into consideration all the circumstances which contribute to the severity of an epidemic—the number seized, and the type of the disease—in no part of England was it marked by the same malignancy of character and the same remarkable prevalence of attack in combination, as in Lincolnshire. In England, as in France, the tendency of the pestilence in its progress was northward. In this respect resembling cholera and influenza, yet unlike them in not showing the same propensity to extend, as they, from east to west. Apparently capricious in its mode of seizure—in its course and in its results, too often feebly controlled by art, it maintained its destructive tendencies, whether these were expressed by typhoid symptoms—by asphyxia, or by a general failure of the powers of life, either immediately or remotely. We will not now stop, to inquire what were the pathological facts developed, and to discuss the various modes of treatment adopted by British practitioners. The consideration of these, we postpone until we shall have glanced at the disease as it has presented itself in this hemisphere. The earliest notice we have of it in this country, is from the pen of Dr. Baird, who, under the title of "suffocative angina," has recorded the particulars of an epidemic that occured in New York in 1771. He describes the pseudo-membranous patches characteristic of this disease. More recently, Dr. Bell observed in Philadelphia an epidemic supposed to be diphtheria. These observations have not been made with the care and exactness that would entitle them to a formal or lengthened notice.

Still more lately, under the head of "Pseudó-membranous Laryngitis," several cases of Diphtheria have been detailed by Dr. Meigs. These cases were seen in Philadelphia in 1845. (American Journal of the Medical Sciences for April, 1847.) In some there was no fever, in others, this was violent. The fauces were covered with fibrinous exudation. The external lymphatic glands were much enlarged. Dr. Meigs' treatment consisted in emetics of alum, general bleeding, calomel, leeches to the throat, tartar emetic, purgatives and warm baths—means which would be wholly inadmissible at present. Nitrate of silver was applied in solution to the fauces. As several of his patients recovered under this treatment, it is reasonable to infer, the constitutional symptoms, were not of the malignant nature they have been in later epidemics. Dr. Meigs approved of alum as an emetic, a teaspoonful of which he administered as a dose, given in molasses as a vehicle, and repeated in ten to twenty minutes, if necessary. During the months of July, August, and September, 1848, Diphtheria appeared as an epidemic, in Augusta. Dr. Henry Campbell, of the Medical College of Georgia, speaks of it in his work, on the "Nervous System in Febrile Diseases." He thus writes: "There is occasionally met, a sporadic case (of Diphtheria;) but the disease occurred here once in the form of a most destructive epidemic, during which, a deplorable number of the children in Augusta and its vicinity fell victims. Paroxysmal fever prevailed that year, perhaps to no unusual extent; but in every child affected with the fever, the, at first, fatal marks of this terrible affection would show themselves. The earlier cases, however, were unattended with any febrile symptoms. The child might appear perfectly well until the very disagreeable odor of the breath, or perhaps some difficulty of respiration would attract the attention of the parent or physician. On looking into the mouth, the posterior part of the pharynx would be found covered with the characteristic white secretion or false membrane; and the case under these circumstances, was often far advanced towards a fatal termination.

"We have said that nearly all the earlier cases were without fever, and yet this was by far the most fatal period of the epidemic. As it progressed, fever presented itself in some of the cases as a complication, and this fever was soon discovered to be of a paroxysmal character."

Dr. Blake considered the enlargement of the cervical glands the most common cause of death. In Dr. Wooster's cases, "the disease," he says, "was recognized by the following characteristics: high fever, rapid pulse, congested and watery eyes, coryza, diminution of urine, difficulty of deglutition, even of fluids; invariably by enlarged tonsils—one or both; by the characteristic pseudo-membranous exudation on the tonsil uvula pharynx or nasal lining."

According to Dr. Pollard, of Richmond, the disease has prevailed recently to a considerable extent in Shirley on the James river in Virginia. About thirty cases occurred, of which two proved fatal. Dr. Pollard's observations are particularly valuable, as they clearly illustrate the inefficacy of calomel in diphtheria. He administered it to a child aged five years, the attack partaking of a mixed character, malignant and croupal, in the dose of 8 grains on the 1st day; on the 2d, 3 grains every 6th hour; on the 3d and 4th days, 3 grains every 4th hour; on the 5th day he suspended it, as "green spinach" discharges made their appearance, and on the 7th day the child died. This and other cases induced him to withdraw his confidence from mercury in diphtheria, and to depend rather on supporting treatment. In the June number of the Boston Medical and Surgical Journal, there is a very practical and useful paper on the disease by Dr. Beardsley, of

Milford, Conn. In it he states that the disease first appeared in Orange, a town, as he remarks, "in an elevated situation, and in a remarkably healthy place, with only a sparse population, and for a while was confined entirely to the scholars attending a select school in the village, but who were boarding in different parts of the neighborhood." This he mentions to show that there was some endemic influence in operation. "Fourteen cases out of fifteen of those who were first attacked, proved fatal, in periods varying from six to twenty-four days. Twelve of these cases were in three families, four in each. Five were under ten, eight were ten to twenty-two, and one was forty years of age."

"Most persons residing in the district where the epidemic first appeared, had at different periods, and during its prevalence, symptoms of the disease, more or less severe; but those who were exposed, and attacked severely, (the writer among this number,) in removing to another locality, suffered from only a modified and mitigated form of it,—corresponding, in this respect, to the general law of endemics." He remarks that "the first symptom of this disease, and it is one," he observes, "which we have never seen referred to by any writer on the subject, was pain in the ear." We think, however, Dr. B. will find in Bretonneau's first case, mention made of this symptom. "It was," he continues, "invariably present, in every case that came under our observation, for a day or two before the patient made the least complaint in any other respect, and before the smallest point or concretion of lymphatic exudation could be discovered on the tonsils or elsewhere."

In Dr. Beardsley's cases, the appetite and digestion were generally unimpaired; and "although typhoid fever was superadded, or ensued in one or two instances, there was not in any case, mental disturbance that was observable, during any stage of the disease." In Dr. Pollard's cases, want of appetite was a very striking characteristic in every form of the complaint. Dr. Beardsley experienced no satisfactory results from external revulsives, and the topical application of astringents. "Hydrochloric acid and tincture of myrrh, combined in equal parts, and applied with a sponge to the fauces, detached the pseudo-membrane more readily, and diminished the liability of its being renewed, more effectually than any other remedy used for that purpose. But Monsell's salt was found to be the most efficacious and valuable of all topical applications;" "and the free use of alcoholic stimulants, especially brandy and whiskey, generally produced profuse diaphoresis, by which great relief was experienced, not only from the general depression of the nervous system, but also from the most formidable local affection."

Since that, we have had the disease in Sacramento in 1856; and we are indebted to Drs. Blake and Fougeaud, for two well written papers on Diphtheria as it occurred there. From Dr. Wooster we have had an instructive essay embodying his views of the disease, as it appeared in San Francisco. Dr. Fougeaud seems to have encountered croupal Diphtheria more frequently than the more malignant form.

From what we have now seen, it may be remarked that this disease has modifications not only in degree, but in form. It has also various modes of accession. It may destroy life in a few hours by a violent and deep impression on the nervous centres attended by congestion of the internal organs; in this form, we may not be able to witness the pseudo-membranous exudation supposed to be pathognomonic of it. What a remarkable analogy there is in this, to what we occasionally see during an epidemic of scarlet fever: particularly in the commencement, when its virulence is most marked. Then it is not very uncommon to meet with a case terminating in death, after from two to four hours illness, without presenting the usual characteristic signs of the disease; a sudden morbid influence on the nervous system, and extreme congestion of internal organs, being all we have to account for the rapidly fatal result. In diphtheria as thus occasionally in scarlet fever, we may have a deadly pallor of the surface—a dusky hue of the countenance, and particularly of the lips, a soft irregular pulse, tongue moist and livid, pupils dilated, drowsiness, urine limpid, and often suppressed, no complaint of local pain, and an air of indifference when roused from stupor. In such cases, death will take place within four hours. This form of diphtheria is rare; but there is another more frequently met with, and scarcely less malignant. The subject of it, generally a child, perhaps retires to rest apparently in its usual health. In the middle of the night, or rather towards morning, it awakes with a sense of distressing nausea, followed by vomiting of a thin whitish glary fluid. Then there is a purging of something similar; the fluid discharged from the stomach and bowels being particularly offensive. The child most probably does not complain of uneasiness in the throat: he is drowsy, and seems disinclined to answer questions. The face is pallid and its expression altered. On examination, we are struck with a shining crimson appearance of the mucous membrane of the fauces. From the velum a tenacious thin sheet of translucent mucous hangs like a curtain over the base of the tongue, the papillæ of which are developed, its surface dry, clean and red. The pulse is rapid, irregular, and compressible. The skin may be warm, though more generally cool. After a lapse of some hours, reaction takes place, and now there is a difficulty of deglutition. Drowsiness is succeeded by delirium; respiration is more frequent. The neck is swollen, hard and tender, chiefly in the parotid and submaxillary regions. The anterior half of the tongue may be clean, but posteriorly it is coated with a thick fur, which sometimes is continued to its tip. The whole of the fauces is covered with a deposit like wash-leather. One or both tonsils swelled. The breath is offensive. A thin sanies issues from the nares and there may be epistaxis and bleeding from the gums. At first, the urine is limpid, but should the attack not terminate in death within twenty-four hours, it becomes more colored and there will be a deposit of lithates; at a later stage it is albuminous and contains the coloring matter of the blood; petechial spots form on the surface: diarrhœa sets in, or if it has been persistent from the beginning, the discharges become altered in appearance, being like what we occaisonally see towards the close of dysenteric cases—serous, and like the washings of flesh, accompanied by intolerable fœtor. The surface grows cold and either coma or a tetanic convulsion terminates life. When diphtheria thus presents itself, it generally proves fatal within four days. Few cases of this kind escape, and fortunately it is not the usual type of diphtheria, even when malignant. The malignant form of the disease most familiar to practitioners, during an epidemic, commences with a sense of lassitude preceding a variable amount of fever and slight soreness of throat. The pulse becomes rapid, small and compressible; the tongue is covered with a thick, yellowish dirty-brown coat; the uvula, velum and pharynx are at first of a dusky red; deglutition is painful and difficult; the neck, about the parotid and submaxillary regions, swells; from the nose, distils an acrid humor; the voice changes; the breath grows fetid; the breathing, from mechanical obstruction, is stertorous; there may be vomiting, and there is much thirst. In some hours the erysipelatous hue of the fauces is replaced by a deposition as if, as Dr. Blount expressed it, a "thin layer of pie-paste was spread over the parts, the edges being thick and abrupt." The urine is scanty and albuminous; debility increases, and the patient sinks exhausted, often retaining to the last, the intellect in its integrity. This is the most frequent mode of accession, progress, and termination of malignant diphtheria; and its duration is generally from eight to ten days. The next variety of the disease is what has been termed croupal. In some epidemics, this has been the prevailing type. It does not appear to be attended with the same oppression of the system of those already described. A sense of constriction in the larynx is an early symptom; the accompanying fever is generally sthenic in character, though some such cases have not presented, from beginning to end any pyrexia—the false membrane rapidly extends to the respiratory organs; there is a hoarse, barking cough, with occasional paroxysms of suffocation, and death takes place by asphyxia. Nothing can contrast more strongly than the peaceful termination in death from asthenia so frequent in malignant diphtheria, and the distressing and painful struggle witnessed when asphyxia thus closes the scene.

In proportion to the suddenness of invasion and the signs of congestion will be the gravity of prognosis; whereas, when the invasion has not only been more gradual, but appeared in the commencement with symptoms of catarrh, it will be favorable. This fortunately is the form in which this disease most frequently appears in this country. Coryza, slight fever with headache and a pain in one or both ears usually usher in the attack. There is some soreness of the throat without swelling either of the fauces or the glands externally. The appetite is often scarcely lessened. There may be diminished energy, but not so much as to prevent the patient attending to his usual pursuits. The fauces on examination, will present one or more insulated patches of pseudo-membranous exudation of a greyish-white appearance, not so defined at the edges nor bordered with the same distinct redness as in the malignant variety, but shaded off, as it were. In this form, which lasts from five to ten days, there may be some albuminuria; and even after the more prominent signs of the disease shall have passed away, albuminuria may for a variable length of time be persistent and accompanied by an anœmic state of the system.

When the febrile symptoms are slight; when there is no stupor; the pulse unaffected; the surface neither cold, nor of a dry burning heat, but soft and moderately warm; when the expression is not altered; the face not pallid nor dusky, the lips retaining their ruddy hue; the neck unswollen; the papillæ of the tongue undeveloped; and instead of a dry erysipelatous, and as it were varnished crimson, appearance of the fauces with much œdema of the woula and soft palate, there is a bright red aud moist surface on which may be discerned the usual vascular arrangement; notwithstanding that the patches may be still adherent but separating at the edges, provided there be no appearance of fresh deposit, then may we hope for a favorable result. But it must be remembered, there is no disease in which amendment is more fallacious. We cannot, either from a particular sign, or assemblage of symptoms, calculate with the same approximation to certainty as in other affections, what the termination may be. The disease may have commenced without any indication of peculiar danger—may observe a course in accordance with the mildness of its accession—there may even be an improvement in every symptom, when suddenly, hope is chilled and the buoyancy and confidence sinks in despair; the patient so lately supposed free from risk, is all at once seized with croupy symptoms, and in a few hours ceases to exist. The deceptive nature of these cases is most happily expressed by a recent writer. (Brown, *Half-yearly Abstract*, vol. XIII p. 70.) "I have seen them die," he says, "in four hours from such sudden invasions; they may linger five or six days, with intermissions of eight or twelve hours; the croupy breathing would suddenly cease; the little sufferer would sit up, smile, eat, drink, and amuse itself. The delighted parents would point to him in admiration of your skill. The sonorous breathing, which told so plainly at your last visit, that death was there, has disappeared; and off your guard, you, in the general joy, pronounce him safe. A few hours suffice to turn this joy into mourning; the stridulous breathing returns, to end only with life." In a case like this, a post-mortem examination will reveal the extension of disease from the pharynx to the respiratory passages, and death will have been the result of asphyxia. But it is by no means necessary to the production of asphyxia that the larynx should participate in the complaint—the enlargement of the cervical glands and of the tonsils, the swollen and coated state of the fauces and obstruction of the nares in combination with altered innervation, can of themselves produce asphyxia. In addition, there is in the malignant form of diphtheria, a more or less congested state of the lungs; in many cases, lobular pneumonia, and thus there is no difficulty in understanding how, in numerous instances of the disease, an exudation of pseudo-membrane in the larynx is not essential to the production of asphyxia.

The French physicians first observed that diphtheria might run its course from beginning to end, to recovery or death without a single pyrectic symptom. And though pyrexia be present, the type of the accompanying fever is not always the same. The tendency usually is to the typhoid. In this country, the attendant fever has occasionally been intermittent. During the epidemic in Augusta in 1848, Dr. Campbell remarking the accompanying fever to be paroxysmal in character, was induced to adopt an antiperiodic medication, which proved more successful in his hands than that previously employed. The disease has also within a short distance of this city been attended with the same form of fever. This may perhaps be owing to the region in which it appeared, being malarious; as it also was most probably the determining cause in Augusta.

The exudation of false membrane exists in all case of diphtheria, except in those extremely rare ones wherein the system is at once overwhelmingly oppressed by the attack, and life is extinguished ere time is given for the usual characteristic phenomena to be fully developed. It is not the cause of any subsequent train of constitutional symptoms, as Bouchut imagined. Neither is it a mere complication. But it is a link in the chain of pathological events—a never failing evidence of a peculiar morbid condition of the general system. Not more real perhaps than others—but one more clearly expressed. As constant as the inflammation and ulceration of the clustered glands in enteric fever—as the rash and catarrh of measles—the sore throat and rash of scarlatina. It appears very early in the disease, within eight or thirty hours from the beginning of the attack, commences generally on one tonsil or rather in the sulcus between the anterior pillar and tonsil, like a stain left by nitrate of silver on a mucous surface—a pearl-colored spot on a red ground. Sometimes it appears, at first, on the uvula or velum. From some one of these points, it more or less rapidly extends, so as often, in from twenty-four to forty-eight hours, to cover the tonsils, uvula, velum, pharynx, pillars of the fauces and base of the tongue, with a continuous layer. It may pass along the respiratory passages to the bronchial tubes and even down the æsophagus; according to some, as far as the cardiac orifice.

Has it ever extended to the stomach and intestines? We are not aware of any post-mortem examination that has revealed it in connection with this disease. Not that the stomach and intestines are insusceptible of an influence under which an exudation of false membrane is occasionally poured out; and that too, both in acute and chronic disease. Andral saw it in chronic gastritis, and Habershon has detailed several cases wherein it was a complication of acute and chronic complaints; but in no instance, as far as we can learn, has it been visible on the mucous surface of the stomach or intestines in connection with the disease we are now considering. Future investigations may show that the exudation is less limited in this direction than pathologists at present imagine. It may pass from the nares along the nasal ducts to the conjunctivæ. The mucous membrane of the cheeks, of the gums; the cutaneous surface, the vulva and the anus may be its seat. Its thickness increases with its area. Its appearance is variable. Sometimes it is in distinct, dull white patches; at others, it is confluent. Occasionally it assumes a light buff, yellowish, ash-colored, or even black appearance. Its thickness varies from the thinnest imaginable pellicle to two or three lines. It may be firm, or be a mere diffluent pulp. The mucous tissue, on which it rests, is, in the fauces, generally unaffected by gangrene or ulceration; not, however, universally so; as one of these states will now and again exist. It is always congested and more or less infiltrated with products of inflammatory action. On its surface, between the basement membrane and epithelium, is the pseudo-membranous deposit exuded. After a few days it begins to be detached, separating at the edges, which curl up, and at the same time an offensive sanies flows from the surface now being exposed. It comes away either by piecemeal, in shreds, or with a more extensive surface. When violently removed, whenever this is possible, the mucous membrane beneath presents numerous bleeding points, but its surface is usually but slightly excoriated. However, when the cheek is the seat of the deposition, on the detachment of this, the surface will be more commonly ulcerated. Examined with the microscope, different appearances are seen, as the portions exuded at first or at a later stage are viewed. That first exuded consists of a net-work of coagulated fibrin mixed up with epithelium cells and with granular matter, whilst that subsequently formed, and in immediate contact with the mucous tissue, is more free from cells and consists of nearly lymph alone. The arrangement is laminar.

It was supposed by Dr. Laycock (*Med. Times and Gazette*), that the disease was the effect of a parasitic fungus (*oïdium albicans*) affecting the surface of the fauces and mouth. This is now thought to be merely accidental, and not necessarily connected with the affection. Some observers state, they can discern a distinct fibrillation; but this is denied by others. It appears incapable of organization, and in this, certainly differs from the matter exuded in croup, in which we have in some instances distinctly observed a well marked vascular arrangement. In stating this, we are aware we differ from Dr. Bristowe, who in the *Med. Times and Gazette* has published an excellent paper on diphtheria, yet we hesitate to receive opinions based merely on microscopic observation. It is not likely that products resulting from two so different conditions of the system as obtain in diphtheria and croup will be identical. The one disease is characterized by an altered state of the whole organism—by asthenic and toxic symptoms; whilst the other, as far as constitutional symptoms are concerned, has not a feature in common with it; and, moreover, in confirmation, admits with advantage of a treatment that has been proved to be positively injurious in the former. About the fourth day we will most probably have swelling of the neck. Now there is something peculiar in the appearance of this swelling. It is not shaped like that seen in scarlatina; it is more oval. It does not efface the angles of the jaws, neither is it so rounded or so equally diffused. The effusion which contributes to it seems to be rather under the deep than the superficial cervical fascia—especially under the layer that immediately covers the sterno-cleido-mastoid muscle. The submaxillary gland is more disposed to enlarge than the parotid and cervical. The swelling of the neck externally appears to be proportioned to the amount of disease in the fauces; where we find the glands much increased in size and generally swollen even down to the clavicle, we need not be surprised to see, on a post-mortem examination, the tonsils not merely enlarged and their sulci filled with purulent matter, but in some few cases that even gangrene has seized on either of them. We need not expect to find nature in the progress of morbid change, limiting herself to the accurately defined path, to which some writers, in their zeal for system and distinctive pathological conditions, would fain restrict her. We will therefore occasionally see in this disease, ulceration and gangrene of the tonsils. When the attack is mild, and the general health unreduced, there is seldom the intolerable odor of the breath experienced when the type is malignant and a tendency to decomposition exists not only in the system itself, but in the morbid products separated from it. Vomiting is a formidable symptom in diphtheria. It declares itself, at two distinct periods. It may usher in the usual train of morbid phenomena, or be the immediate precursor of a fatal termination.

We have now viewed diphtheria as it destroys life or finally disappears, after a few weeks at farthest; but it may be seen under other phases. The patient may have recovered from the immediate effects of the attack; he may even appear convalescent; and yet, without any further well-defined symptom, he may sink in several weeks after all the characteristics of diphtheria shall have disappeared; or he may suffer from partial or general paralysis. Paralysis may not set in as a sequela for some weeks after the date of the seizure of the complaint. After all disappearance of false membrane, and when deglutition is no longer painful, a morbid impression still continues to be made on the nervous tissue, causing not merely a persistence of general depression, but even impairing or altogether suspending the functions of the nervous system. Four cases are related by Dr. Eade, of Norwich, in which paralysis supervened on diphtheria: in the first case the person began to complain of paralysis in a month after apparent recovery from diphtheria; in the second, in fourteen days; in the third, in ten weeks; and the fourth case is that of a patient in whom the original attack was so slight as not to prevent his working. In a fortnight afterwards he thought he was quite well, when he was seized with numbness in his hands and feet, preceded for two or three days, but not accompanied, by vague pains in the back and elsewhere. (Cases of Paralysis, by Peter Eade, M. D., *Lancet.*) In all these cases, the spinal system was especially engaged. In two, the fifth nerve was affected, there being numbness of the cheeks and nose.

Whilst our attention is directed to this part of our subject, we cannot avoid considering somewhat in detail the phenomena more immediately dependent on the undoubted though mysterious influence of some poison on the nervous system. We have been struck with the generally unmanageable nature of the cases in which violent and long-continued vomiting has been a prominent symptom, and have been induced to consider this as the result of a direct and morbid impression on the source of the par vagum. To this we also refer much of the dyspnœa encountered in some cases, wherein neither the amount of pseudo-membranous deposit, nor the situation of it, nor the extent of external swelling of the neck, nor of the fauces can satisfactorily account for this symptom. Perhaps Dr. Pollard's 2d case, as related in the *Virginia Medical Journal*, for September, 1859, will supply what is illustrative of this. The dyspnœa and vomiting in that case were most prominent symptoms, and will admit of explanation on this ground more readily than on others. In many of the untoward cases the fatal result has been preceded by a loss of deglutition, which should rather be referred to the same source than to the mere injury done the muscular tissue itself. It is probably due to impairment of the motor functions of the pharyngeal branches of the par vagum: perhaps, also, the muscular branches of the glosso-pharyngeus, which supply the stylo-pharyngeus, and partially, the constrictor muscles, receive some unfavorable impression of the same kind. The afferent functions of this nerve may likewise be compromised; the conveyance of impressions from the surface of the fauces to the medulla oblongata being suspended by the noxious influence of the poison on the nerve. These paralytic affections that appear as sequelæ of diphtheria, almost always yield to suitable treatment. A case, however, is recorded where death took place from a lesion of the nervous system expressed during life by hemiplegia. Cases of amaurosis are also recorded, owing their origin to the same cause.

Diphtheria, when epidemic, is also contagious. There is no fact better ascertained than this. A child with the disease was attended by M. Herpin, of Tours. During one of his visits, the patient being seized with a fit of coughing, some of the diphtheritic matter that was ejected having rested on the aperture of M. Herpin's nostril, he for some time omitted to remove it. The result was near being fatal. Diphtheria declared itself, which, being cured with difficulty, was followed by a protracted convalescence. Gendron, of Chateau de Loire, received the disease in a similar manner, and narrowly escaped. In addition, a case is recorded of a boy having contracted cutaneous diphtheria by using a bath in which a diphtheritic patient had been previously bathing.

The post-mortem examination of those who have died of diphtheria, often reveals serious lesions. In severe cases, the areolar tissue of the neck will be not only congested, but infiltrated with a yellow gelatinous serum, or even with blood; and this frequently from the jaw to the clavicle. The pharynx, and, for some distance, the œsophagus, the tonsils, the cheeks internally, the mucous membrane of the nares, and its prolongation towards the eye, the pillars of the fauces, and both surfaces of the epiglottis, velum, uvula, larynx, trachea, and bronchial tubes are occasionally covered with the characteristic pseudo-membrane. The sacculi laryngis may be filled up with it. The mucous membrane beneath may be but slightly excoriated; it is not often ulcerated, and still less frequently gangrenous. The tonsils may be simply congested, may contain suppurating cavities, or even be gangrenous. In the worst cases, the lungs will present patches of red hepatization or of purulent infiltration separated from each other by portions of crepitant lung. Elsewhere there may be more or less of simple congestion, and the bronchial tubes may contain much mucous. Blood is seen occasionally extravasated in the muscular tissue of the heart. In malignant diphtheria the kidneys often bear marks of disease; though in every case of this disease wherein albuminuria exists, we do not attribute it to a positive change of structure. In some cases, there will be seen hemorrhagic exudation into the tubules, and a granular condition of the epithelial cells. Sometimes the channel of the tubes is lifted up by them, and transparent fibrinous casts are seen. The viscera are often dotted over with petechial spots, owing, most probably, to some change having taken place in the blood.

(To be contiuued.)

---

